# Development and evaluation of a prototype non-woven fabric filter for purification of malaria-infected blood

**DOI:** 10.1186/1475-2875-10-251

**Published:** 2011-08-25

**Authors:** Zhi-Yong Tao, Hui Xia, Jun Cao, Qi Gao

**Affiliations:** 1 Department of Parasitology, Bengbu Medical College, 2600 Donghai Dadao Road, Bengbu 233030, People's Republic of China; 2 Jiangsu Institute of Parasitic Diseases, Meiyuan Yangxiang 117, Wuxi 214064, People's Republic of China; 3 Key Laboratory on Technology for Parasitic Disease Prevention and Control, Ministry of Health, Meiyuan Yangxiang 117, Wuxi 214064, People's Republic of China; 4 Department of Parasitology, Medical College of Soochow University, Suzhou 215123, People's Republic of China

## Abstract

**Background:**

Many malaria-related studies depend on infected red blood cells (iRBCs) as fundamental material; however, infected blood samples from human or animal models include leukocytes (white blood cells or WBCs), especially difficult to separate from iRBCs in cases involving *Plasmodium vivax*. These host WBCs are a source of contamination in biology, immunology and molecular biology studies, requiring their removal. Non-woven fabric (NWF) has the ability to adsorb leukocytes and is already used as filtration material to deplete WBCs for blood transfusion and surgery. The present study describes the development and evaluation of a prototype NWF filter designed for purifying iRBCs from malaria-infected blood.

**Methods:**

Blood samples of *P. vivax *patients were processed separately by NWF filter and CF11 column methods. WBCs and RBCs were counted, parasite density, morphology and developing stage was checked by microscopy, and compared before and after treatment. The viability of filtrated *P. vivax *parasites was examined by *in vitro *short-term cultivation.

**Results:**

A total of 15 *P. vivax*-infected blood samples were treated by both NWF filter and CF11 methods. The WBC removal rate of the NWF filter method was 99.03%, significantly higher than the CF11 methods (98.41%, *P *< 0.01). The RBC recovery rate of the NWF filter method was 95.48%, also significantly higher than the CF11 method (87.05%, *P *< 0.01). Fourteen *in vitro *short-term culture results showed that after filter treatment, *P. vivax *parasite could develop as normal as CF11 method, and no obvious density, developing stage difference were fund between two methods.

**Conclusions:**

NWF filter filtration removed most leukocytes from malaria-infected blood, and the recovery rate of RBCs was higher than with CF11 column method. Filtrated *P. vivax *parasites were morphologically normal, viable, and suitable for short-term *in vitro *culture. NWF filter filtration is simple, fast and robust, and is ideal for purification of malaria-infected blood.

## Background

Malaria is a mosquito borne parasitic disease that causes about 1 million fatalities a year, most occurring in Africa. According to a WHO estimate, 3.3 billion people are at risk of malaria [1]. Because no effective vaccine currently exists, all humans are potentially susceptible to malaria. Better control and eradication of malaria requires more thorough research on the biological characteristics of the parasite.

The establishment of continuous cultures of *Plasmodium falciparum *provided researchers with abundant materials that led to several breakthroughs [2]. Compared to *P. falciparum*, research on *Plasmodium vivax *is less extensive, not only because vivax malaria is relative mild and therefore neglected, but also because of the biological properties of *P. vivax*[3]. First, *in vitro *cultivation of *P. vivax *is not well established, and limited to short-term maintenance. Usually, parasite density of *P. vivax *during culture does not obviously increase as it does with *P. falciparum*, but decreases [4]. Second, the animal model of *P. vivax *is endangered non-human primates, which are difficult to obtain. The biological properties of *in vivo *propagated parasites are not exactly the same as parasites isolated from naturally infected humans, so they cannot represent the entire set of characteristics of *P. vivax *in nature. Rodent malaria parasites are easy to propagate *in vivo*, and parasitaemia can be high, making it a realistic animal model for some malaria researches [5]. However, blood from infected human or animal models includes leukocytes, and especially in cases of *P. vivax *cases, the amount of WBCs is often equal to or greater than iRBCs. These host source WBCs have high metabolism capacity, are a contamination in malaria biology, immunology and molecular biology studies, and need to be removed [6].

Currently, the methods for removing WBCs from malaria-infected blood include CF11 column and Plasmodipur filter filtration [7-13]. CF11 cellulose column is the most commonly used method, it is cheap but time consuming and laborious, and Plasmodipur filter is expensive for resource-limited settings, therefore a new method is required for purification of malaria-infected blood. Leukocytes can be depleted by filtering through non-woven fabric filters [14], because this type of blood cells had strong adhesion function based on its cell surface properties. This is in contrast to RBCs, which are capable of deformation, and can escape from the rigid fibers [15]. Even though the surface of iRBCs is altered by parasite protein trafficking to the host cell membranes, iRBCs still flow through the filter material, allowing iRBCs to be purified from WBC contaminants. The present study describes the development and evaluation of a prototype NWF filter designed for purifying iRBCs from malaria-infected blood.

## Methods

### The development of prototype NWF filter

The PBT (polybutylene terephthalate) melt-blown non-woven fabric modified with polar groups(Gaolite medical equipment Ltd., Zhangjiagang, China) was used as the filter media, and the shape of NWF filter was referred to single-use sterilizing filter. Injection molding machine (MA1200/370, Haitian, Ningbo, China) and ultrasonic plastic welder (EM20-2, Branson, Shanghai, China) were used to produce and assemble the polycarbonate shells with NWF filter pad.

There are four major criteria of new filter for purification of malaria-infected blood: a) One single-use filter is capable for treatment of 5 ml *P. vivax*-infected blood; b) For 5 ml whole blood, the WBC removal rate should be >99%; c) After filtration, malaria parasite keeps viable, and is suitable for *in vitro *short-term culture; d) It is also suitable for treatment of rodent malaria-infected blood.

### *Plasmodium vivax-*infected sample collection and processing

*Plasmodium vivax*-infected blood samples were collected in the north part of Anhui province from July 2007 to September 2010. Only *P. vivax *was prevalent in this area [16]. All malaria cases were confirmed by microscopy. After consent was obtained from adult patients, 10-15 ml of whole blood was drawn into heparin-treated vacuum tubes, and mixed. Blood samples were sealed and immersed in 37°C water in a thermos flask and immediately transferred to the laboratory in the Parasitology Department of Bengbu Medical College. Thick- and thin-smear were made, stained with Giemsa (Fisher, USA), and examined by microscopy. After mixing and transferring whole blood into a 50 ml centrifuge tube, and spinning for 5 min at 2,000 rpm, plasma was removed. To compare efficiency of leukocyte removal, incomplete RPMI 1640 medium (Invitrogen, USA) was used to resuspend the cell pellet to 30 ml. A 200 μl cell suspension was transferred to a centrifuge tube, for counting WBCs and RBCs using the improved Neubauer chamber method. A 20 ml cell suspension was separated in two 50 ml centrifuge tubes, each containing the equivalent of about 5 ml of whole blood.

### Purification of *P. vivax*-infected blood sample by the NWF filter method

Ten ml of cell suspension prepared above was sucked into a 20 ml syringe, the filter inlet interface was adapted to the syringe, and the plunger was gently pushed (about 5 ml/min). Then collect flow-through cell suspension from the filter outlet into a new 50 ml centrifuge tube. The filter was washed with 5 ml RPMI 1640, and eluted cell suspension collected in the same tube. The sample was spun 10 min at 3,000 rpm, the supernatant discarded, and RPMI 1640 used to resuspend the pellet to 10 ml, and WBCs and RBCs were counted as described above. A 10 μl cell suspension was used to make thick- and thin- smears for staining and examination as above.

### Purification of *P. vivax*-infected blood sample by CF11 column method

CF11 powder was purchased from Whatman (Maidstone, UK), and CF11 columns were prepared as described previously [7]. The CF11 powder bed was packed in a central luer syringe column with a sterile foil package by tapping on the bench, and a pre-rinsed CF11 bed was made by dropping in 5 ml RPMI 1640. Before the last portion of liquid ran into the bed, 10 ml of cell suspension was applied to the bed dropwise, and then the bed was rinsed with 5 ml RPMI 1640. The cell suspension was eluted into a 50 ml centrifuge tube. Sample washing, counting of WBCs and RBCs, slide making and checking were as above. And the results were compared to NWF filter method.

### *In vitro *short-term culture of filtrated *P. vivax *samples

Both NWF filter and CF11 column treated RBC samples from *P. vivax *patients with more than 50% ring stage parasites and without prior anti-malarial therapy were washed three times with RPMI 1640, and resuspended at 2% haematocrit in McCoy's 5A medium (Sigma-Aldrich) containing 20% human AB+ serum (prepared from malaria-naive donors)[17] and added into 10 wells of a 96-well plate, each well contains 100 μl of cell suspension. The test plate was incubated at 37°C, 5% CO_2_, humidified environment for 48 h. A thick- and thin- smear was made to check the development status of *P. vivax *parasites for every 8 hours. The parasitaemia and morphology of parasite of each slide were checked by microscopy.

### Purification of *P. berghei*-infected mouse blood samples

Heparin-treated *Plasmodium berghei*-infected mouse blood samples were provided by the Parasitology Department of Bengbu Medical College. Plasma of each sample was removed by centrifugation, and packed cells were diluted in RPMI 1640 to 5 ml, then filtered through NWF filter and washed as previously for *P. vivax *samples. Sampling and counting for WBCs were carried out as above. Thick- and thin-smears were made and Giemsa stained pre- and post filtration for observation.

### Purification of *P. falciparum *sample *in vitro*

A 0.5 ml of *in vitro*-cultured *P. falciparum *(FCC1/HN isolate) sample at 5% parasitaemia was mixed with fresh prepared 4.5 ml of healthy volunteer's plasma-free blood, and then filtered through NWF filter as described above. Thick- and thin- smears were made and Giemsa stained to observe the change of WBCs and infected RBCs pre- and post filtration.

### Statistical analysis

All data were entered and analysed using SPSS for Windows v14 (SPSS Inc., Chicago, IL). The Wilcoxon two-sample paired signed rank test was used to compare the differences in WBC removal, RBC recovery and parasite density between NWF filter and CF11 column methods. Statistical significance was set at *P *≤ 0.05.

### Ethical considerations

The study was approved by the Institutional Review Board (IRB00004221) of Jiangsu Institute of Parasitic Diseases, Wuxi, China.

Questionnaire surveys, physical examination and laboratory work were conducted after the purpose of the study had been explained to participants, who were given the right to withdraw from the study at any time, without consequences. Written informed consent was obtained from each participant.

## Results

### Development of NWF filter for purification of malaria-infected blood

**Figure 1:**
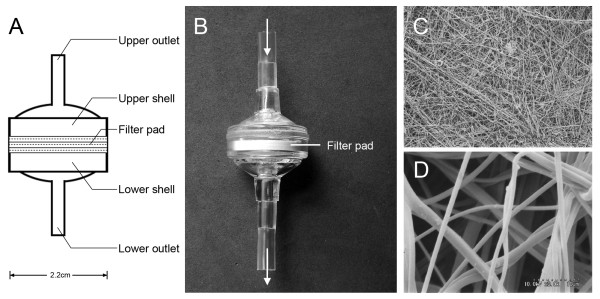
**Prototype NWF filter for purification of malaria-infected blood**. The prototype single-use NWF filter is designed for removing 99% leukocytes from 5 ml malaria-infected blood sample at 95% RBC recovery rate: (A) Diagram of the prototype filter, (B) The shape of NWF filter, (C~D) Scanning electron micrographs of PBT melt-blown non-woven fabric material.

Upper and lower parts of polycarbonate shells of new NWF filter were produced by plastic injection molding. It has three layers of non-woven fabric unit in 2.2 cm diameter as filter pad, and ultrasonic welding method was adopted for assembling the upper part, filter pad and lower part into a prototype NWF filter (Figure 1). Filters were washed by distilled water and sterilized with ethylene oxide gas, and stored in GMP environment for one month before use.

### Comparison of NWF filter filtration and CF11 column methods for purification of *P. vivax*-infected blood

**Figure 2:**
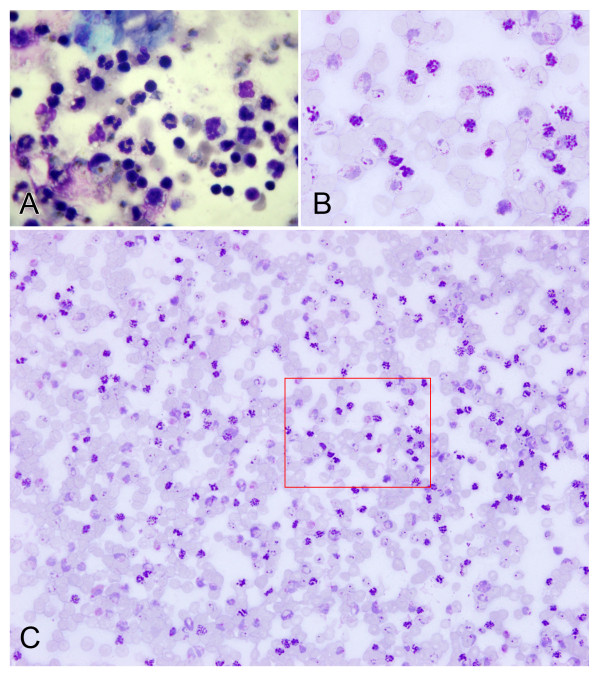
**Leukocyte removal efficacy of NWF filter filtration coupled with 60% Percoll concentration**. A *P. vivax *blood sample was treated by NWF filter filtration then performed 60% Percoll concentration: (A) Excessive WBC contamination in the 60% Percoll enrichment slide without removing WBC (100× oil-immersion objective), (B) With NWF filter filtration, no WBCs could be observed. (100× oil-immersion objective), (C) Even including more RBCs, still no single WBC was fund (40× high power objective), the rectangle represent the same field of (B).

A total of 15 *P. vivax*-infected blood samples were used for both NWF filtration and CF11 methods. WBCs and RBCs were counted before and after treatment, and the removal rate of WBCs and the recovery rate of RBCs were calculated, and the parasite density was counted by microscopy (Additional file 1). The average WBC removal rate by the NWF filter was 99.03%, and the RBC recovery rate was 95.48%. The average WBC removal rate of the CF11 method was 98.41%, and the RBC recovery rate was 87.05%. Significance differences were observed between the two methods for WBC removal (*P *< 0.01), and a clear significant difference between the two methods was seen for RBC recovery (*P *< 0.01). The NWF filter method was better than the CF11 column method, especially for high performance RBC recovery. Post-filtration, there is no parasite density difference between NWF filter and CF11column methods (*P *> 0.05) by microscopy, but the WBCs were substantially reduced (Figure 2).

### *In vitro *short-term culture of *P. vivax *after filtration

**Figure 3:**
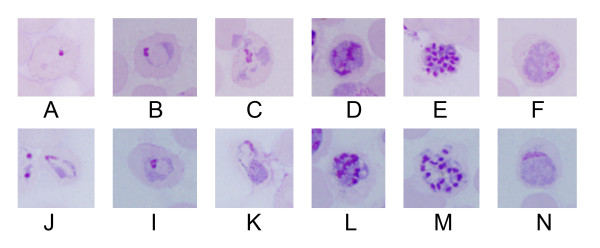
***In vitro *short-term culture of *P. vivax *after NWF filter filtration**. CF11 column method panel (A~F) and NWF filter method panel (J~N). A, J: Rings; B, I: early trophozoite; C, K: late trophozoite; D, L: Young schizont; E, M: mature schizont; F, N: gametocyte.

A total of 14 *P. vivax-*infected blood samples were treated by both NWF filter filtration and CF11 column methods, and subjected to *in vitro *short-term culture. Compared to CF11 column method, parasites filtrated by NWF filter are of same ability to continue developing into mature stage *in vitro*, and there are no obvious changes been observed in parasite density, morphology or stage proportion between two methods by microscopy (Figure 3).

### Purification of *P. berghei*-infected sample

**Figure 4:**
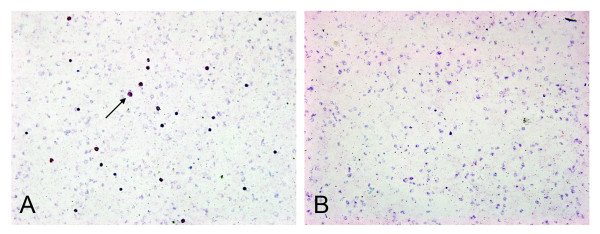
**NWF filter filtration for purification of *Plasmodium berghei***. (A) Before filtration, a great many WBCs were observed (40× high power objective), (B) After filtration, no WBC were observe d (40× high power objective).

Five *P. berghei*-infected mouse blood samples were diluted and filtrated by NWF filter method, the WBC removal rate was 99.21%. By microscopy comparison of pre- and post filtration smears: there are no obvious parasite density, morphology or stage proportion changes of *P. berghei *been observed (Figure 4).

### Purification of *P. falciparum in vitro*

When the WBCs were removed from the sample mixed with *in vitro *cultured *P. falciparum*-infected RBCs and healthy donor blood, there were no obvious changes in parasite density, morphology or stage proportion of *P. falciparum *observed between pre- and post-filter filtration.

## Discussion

The prototype NWF filter developed here was designed to remove >99% of leukocytes from 5 ml of whole blood, with a 95% RBC recovery rate. After testing with 15 *P. vivax*-infected blood samples, the rates of WBC removal and RBC recovery for the NWF filter method were 99.03% and 95.48%, and for the CF11 method they were 98.41% and 87.05%. NWF filter showed both higher WBC removal and RBC recovery rates over CF11 column method. To save time in sample processing, only one wash step was used, which may have contributed to the difference in RBC recovery between the two methods.

In some studies, the purity and viability of parasites in malaria samples are crucial for results, and a convenient method for removal of contaminating WBCs is needed. In this study, processing 5 ml of whole blood using the CF11 method requires at least 30 minutes from pre-rinsing the column bed to the final drop of the cell suspension flow-through. In contrast, the NWF filter method saves time and is easy to perform. Filtration of same amount blood is possible in 5 minutes from diluting sample to the end of the wash procedure. Furthermore, the filtration method is more robust than the CF11 method; the latter method may fail if the column was not prepared properly, resulted in an unacceptable amount of WBCs leakage. NWF filters could be sealed in plastic bag and batch sterilized thus could be ready to use, with no need to prepare or autoclave before use, as is required for CF11 columns. Training for using NWF filter is minimal, and researchers can successfully perform filtration by following the protocol. Filter filtration does not need additional equipment, so it is suitable for use in field settings.

According to malaria genome and transcriptome studies, contamination from the host is the difficult problem in research, lowering the credibility of sequencing and annotation results [18]. For example, about 10% of host sequences are incorrectly in the *Plasmodium yoelii *genome results [19]. By NWF filtration, more than 99% leukocytes were removed from malaria-infected blood, providing purified materials for downstream malaria research, it suggest that NWF filter could be useful for sample pretreatment in malaria molecular studies.

In recent years, reports on the chloroquine resistance of *P. vivax *have gradually increased, and *in vitro *tests were considered as direct and more important evidence [20]. The existence of WBCs in short-term cultures of *P. vivax *not only disturbs the development of parasites and interferes with microscopy, more importantly, makes drug accumulated and decreases the concentration of drugs, and so IC_50 _results may be falsely achieved [21]. Thus, removal of WBCs from *P. vivax*-infected blood samples is a necessary procedure for *in vitro *drug susceptibility tests. In this study, *P. vivax *parasites filtered by NWF filter were successfully short-term cultured *in vitro*, and with no obvious changes in density, morphology and stage proportion compared to CF11 method. Escaped WBCs and platelets may cast negative impact on *P. vivax *during *in vitro *culture, and post NWF filter treatment, the major component of escaped WBCs were lymphocytes, and most of platelets were removed, these properties were similar to CF11 method [7]. The development of NWF filter provides researchers a useful tool for further *in vitro *antimalarial drug sensitivity surveillance.

Using rodent models to investigate malaria has obvious advantages including easy for maintaining, cost efficient and avoiding risks associated with human material. In this study, results showed NWF filter are suitable for purification of *P. berghei*-infected mouse blood, among high WBC removal ability, more importantly, the filter method enables a higher RBC recovery rate than the CF11 method, making it more convenient for treatment of small amount samples. Even *in vitro *cultivation could provide purified *P. falciparum *parasites, though the parasites isolated from patient blood still play unique role in many studies. The new NWF filter could be used for purification of *P. falciparum *samples too. In this study, including mature stage *P. falciparum*-infected RBC could pass through the non-woven fabric filter material, this result is different from the report of using routine transfusion filter to deplete *P. falciparum-*infected RBC [22], this may be caused by the difference of filtration materials, sample size and iRBC load.

## Conclusions

NWF filter filtration removed most leukocytes from malaria-infected blood, and the recovery rate of RBCs was higher than with CF11 column method. Filtration could enable more efficient use of malaria samples for research. No obvious parasite density decrease or morphology changes were observed, and the viability of parasites was also well preserved during processing. *P. vivax *blood samples filtered by NWF filter were successfully used in short-term *in vitro *cultivation. It is also suitable for purifying *P. berghei*-infected mouse blood and *P. falciparum*-infected human blood. A new prototype NWF filter was successfully developed, and the NWF filter filtration method is simple, fast and robust, and ideal for purification of malaria-infected blood.

## Competing interests

The authors declare that they have no competing interests.

## Authors' contributions

ZYT, JC and QG conceived the study and participated in its design and coordination. ZYT carried out NWF filter designing and comparison of filter filtration and CF11 method. HX contributed expertise in field sample collection, *in vitro *short-term culture. ZYT wrote the manuscript. JC, HX helped on results analysis and manuscript drafting. All authors read and approved the final manuscript.

## Supplementary Material

Additional file 1 **The efficacy comparison results of NWF filter filtration and CF11 column methods for purifying *Plasmodium vivax*-infected blood**. The WBC, RBC and parasite density results of *Plasmodium vivax*-infected blood samples before and post treatment by NWF filter filtration and CF11 column methods. Click here for file
