# Supplementary material for: Development and evaluation of a prototype non-woven fabric filter for purification of malaria-infected blood
Source: Malar J. 2011 Aug 25;10:251. doi: 10.1186/1475-2875-10-251 (PMC3173400; doi:10.1186/1475-2875-10-251)
Supplement: Additional file 1 — The efficacy comparison results of NWF filter filtration and CF11 column methods for purifying Plasmodium vivax-infected blood. The WBC, RBC and parasite density results of Plasmodium vivax-infected blood samples before and post treatment by NWF filter filtration and CF11 column methods. [file 1475-2875-10-251-S1.DOC]

**Additional File 1**

**Table 1.** **The efficacy comparison of NWF filter filtration and CF11 column methods for purifying *Plasmodium vivax***-infected blood

| Sample ID | WBC removal | | | | | RBC recovery | | | | | Parasite Density (parasites/10,000 RBC ) | | |
| --- | --- | --- | --- | --- | --- | --- | --- | --- | --- | --- | --- | --- | --- |
|  | Before | NWF filter | | CF11 column | | Before | NWF filter | | CF11 column | | Before | NWF filter | CF11 column |
|  |  | After  (×10^3^/μl) | Removal rate (%) | After  (×10^3^/μl) | Removal rate (%) |  | After  (×10^6^/μl) | Recovery rate (%) | After  (×10^6^/μl) | Recovery rate (%) |  |  |  |
| 1 | 4.33 | 0.03 | 99.31 | 0.04 | 99.08 | 4.36 | 4.21 | 96.56 | 3.86 | 88.53 | 15 | 14 | 14 |
| 2 | 6.87 | 0.03 | 99.56 | 0.03 | 99.56 | 3.79 | 3.63 | 95.78 | 3.23 | 85.22 | 27 | 28 | 27 |
| 3 | 5.36 | 0.05 | 99.01 | 0.07 | 98.69 | 3.21 | 3.03 | 94.39 | 2.81 | 87.54 | 34 | 33 | 35 |
| 4 | 4.79 | 0.02 | 99.60 | 0.05 | 98.96 | 4.16 | 3.98 | 95.67 | 3.62 | 87.02 | 82 | 80 | 78 |
| 5 | 9.51 | 0.06 | 99.37 | 0.10 | 98.95 | 3.59 | 3.47 | 96.66 | 3.29 | 91.64 | 22 | 23 | 22 |
| 6 | 4.52 | 0.04 | 99.12 | 0.07 | 98.45 | 3.72 | 3.57 | 95.97 | 3.17 | 85.22 | 12 | 12 | 11 |
| 7 | 8.26 | 0.12 | 98.55 | 0.17 | 97.94 | 3.28 | 3.16 | 96.34 | 2.85 | 86.89 | 14 | 13 | 15 |
| 8 | 5.28 | 0.05 | 99.05 | 0.08 | 98.48 | 3.27 | 3.09 | 94.50 | 2.75 | 84.10 | 23 | 22 | 24 |
| 9 | 5.98 | 0.10 | 98.33 | 0.11 | 98.16 | 3.98 | 3.85 | 96.73 | 3.59 | 90.20 | 27 | 25 | 27 |
| 10 | 4.33 | 0.05 | 98.78 | 0.13 | 97.00 | 3.38 | 3.23 | 95.56 | 2.89 | 85.50 | 41 | 39 | 40 |
| 11 | 7.76 | 0.11 | 98.58 | 0.18 | 97.68 | 3.13 | 2.93 | 93.61 | 2.62 | 83.71 | 19 | 21 | 22 |
| 12 | 3.27 | 0.03 | 99.17 | 0.08 | 97.55 | 4.47 | 4.25 | 95.08 | 3.82 | 85.46 | 39 | 38 | 37 |
| 13 | 3.36 | 0.03 | 99.17 | 0.06 | 98.21 | 3.26 | 3.14 | 96.32 | 2.85 | 87.42 | 22 | 25 | 24 |
| 14 | 5.37 | 0.04 | 99.26 | 0.07 | 98.70 | 4.09 | 3.86 | 94.38 | 3.69 | 90.22 | 36 | 35 | 37 |
| 15 | 6.41 | 0.07 | 98.88 | 0.12 | 98.13 | 3.58 | 3.37 | 94.13 | 3.07 | 85.75 | 22 | 20 | 21 |
| Average |  |  | 99.03 |  | 98.41 |  |  | 95.48 |  | 87.05 |  |  |  |
